# New evidence for the pharmacological intervention promoting neurorecovery after stroke: results from the joint EAN-EFNR guidelines

**DOI:** 10.25122/jml-2021-1003

**Published:** 2021

**Authors:** Dafin Muresanu, Dragos Cretoiu

**Affiliations:** 1. “Iuliu Hatieganu” University of Medicine and Pharmacy, Cluj-Napoca, Romania; 2. “RoNeuro” Institute for Neurological Research and Diagnostic, Cluj-Napoca, Romania; 3. Carol Davila University of Medicine and Pharmacy, Bucharest, Romania

In this editorial, we highlight the development process and some results from a valuable addition to the landscape of evidence-based medicine in the field of stroke neurorecovery – *the European Academy of Neurology and European Federation of Neurorehabilitation Societies guideline on pharmacological support in early motor rehabilitation after acute ischaemic stroke* [1].

The ethos of this endeavor was to devise a clinical practice guideline on pharmacological support in early motor rehabilitation using a rigorous approach, following methodological gold standards, and focusing on outcomes that are patient-important and useful for clinicians. Ultimately, the goal of this living paper is to support clinical decision-making by any health care professional involved in early neurorehabilitation. Despite significant strides made by neurorehabilitation for motor function after stroke, clinicians have very few instruments to inform clinical decision-making in this area of great burden to economies and society at large [2].

To address this gap, the objective of this project was to determine whether pharmacological treatment impacts early motor performance (1 and 3 months after stroke), neurological function (1 and 3 months after stroke), global functional outcome (1 and 3 months after stroke) or safety (serious adverse events) in patients after acute ischaemic stroke, as compared with standard/usual care. The clinical question is available in the **P**atient/**P**roblem, **I**ntervention, **C**omparison and **O**utcome (PICO) format (Table 1). The core feature of this approach is the 7-day intervention initiation window, which was informed by the *Agreed definitions and a shared vision for new standards in stroke recovery research: The Stroke Recovery and Rehabilitation Roundtable taskforce*, developed by Bernhardt *et al*. in 2017 [3].

**Table 1:** PICO of the European Academy of Neurology and European Federation of Neurorehabilitation Societies guideline on pharmacological support in early motor rehabilitation after acute ischaemic stroke.

Patient/problem:Acute ischemic stroke	**Intervention:**Pharmacological intervention in the first 7 days after stroke
**Comparison:**Neurorehabilitation alone
**Outcomes (N=4):**early motor performance, neurological function, global functional outcome, safety
**Setting:**Early motor rehabilitation after acute ischemic stroke

Guideline development followed the Grading of Recommendations Assessment, Development and Evaluation (GRADE) Working Group, in line with the 2015 recommendations for the preparation of neurological management guidelines by EAN task forces (TFs). The GRADE approach is based on a sequential assessment of the quality of evidence, followed by an assessment of the balance between advantages and disadvantages, and finally, judgment about the strength of recommendations. With help from health professionals and methodologists from six European countries, the guideline Task Force identified outcomes of critical importance for clinical decision-making in all areas of PICO.

Some of the methodological rigors undertaken in the guideline production process, including novelties in the field, include:

- Complete documentation of the systematic search process at the single item level, search terms, capturing each decision in the process to minimize selection bias, promote reproducibility, transparency, and traceability;
- Development of a prospective operational manual for all statistical procedures to be applied in a consistent manner throughout all included interventions;
- Before initiating consultation and appraisal processes following GRADE standards, the guideline production TF decided to raise the level of ethical compliance, adding an additional layer of disclosure related to potential intellectual conflict of interest (e.g., documenting any study-related activities), using forms developed in collaboration with the European Federation of Neurorehabilitation Societies and communicated extensively to all interested stakeholders. TF members have abstained from voting on interventions reported in the above-mentioned disclosure process (normally, only financial disclosures were made within EAN guideline production group forms);
- Development of a fully anonymous voting process according to the Delphi principles, with the technical implementation of auto-mated and secured, private code voting forms (Figure 1).

**Figure 1:**
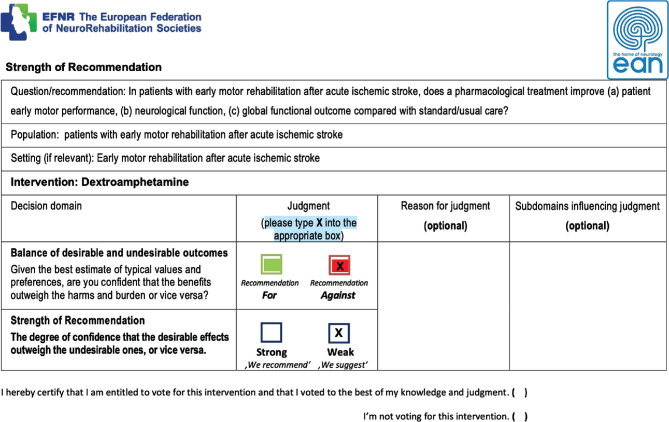
Example of the anonymous voting form used by the guideline production Task Force.

Based on the guidelines summary of results in the context of early motor post-stroke rehabilitation (within 7 days after onset, only two interventions – Cerebrolysin, 30 ml/day, intravenous, minimum 10 days and citalopram, 20 mg/day, oral – are recommended for clinical use for early neurorehabilitation after acute ischaemic stroke. The remaining interventions identified by our systematic search are not recommended for clinical use: amphetamine (5, 10 mg/day, oral), citalopram (10 mg/day, oral), dextroamphetamine (10 mg/day, oral), Di-Huang-Yi-Zhi (2×18 g/day, oral), fluoxetine (20 mg/day, oral), lithium (2×300 mg/day, oral), MLC601(3×400 mg/day, oral), phosphodiesterase-5 inhibitor PF-03049423 (6 mg/day, oral). No recommendation ‘for’ or ‘against’ is provided for selegiline (5 mg/day, oral). Issues with safety and tolerability were identified for amphetamine, dextroamphetamine, fluoxetine and lithium. Forest plots for all interventions are available in the supplemental data file.

Guideline authors conclude that further efforts are required to provide more precise recommendations and insight into practical aspects to be considered when approaching supportive therapies for early motor rehabilitation after ischaemic stroke. An additional consideration pointed out by this TF is that more effort must be put into building consensus on the agreed types of rehabilitation protocols to be studied. To answer more specific questions regarding frequency, type, or setting for this intervention, new clinical trials must report more detailed information regarding rehabilitation protocols and ideally harmonize protocols based on existing best practices.

Informing clinicians regarding existing pharmacological support in interventions for neurorecovery after acute ischaemic stroke will definitely lead to improved patient outcomes and contribute to alleviating the immense global burden of the disease. We embrace this guideline as an example for future endeavors and look forward to promising advances for the future of post-stroke neurorehabilitation.

## Supplementary Material


