# Supplementary material for: New evidence for the pharmacological intervention promoting neurorecovery after stroke: results from the joint EAN-EFNR guidelines
Source: J Med Life. 2021 May-Jun;14(3):295–7. doi: 10.25122/jml-2021-1003 (PMC8321620; doi:10.25122/jml-2021-1003)
Supplement: Supplementary file 1 [file JMedLife-14-295-s001.pdf]

Figures

Figure 1 (Analysis 1.1)

Study or Subgroup	Experimental Mean	Experimental SD	Experimental Total	Control Mean	Control SD	Control Total	Weight	Mean Difference IV, Fixed, 95% CI
SPRIGG 2007	35.9	26.4	17	33.5	28.2	16	100.0%	2.40 [-16.27, 21.07]
**Total (95% CI)**			**17**			**16**	**100.0%**	**2.40 [-16.27, 21.07]**

Heterogeneity: Not applicable
Test for overall effect: Z = 0.25 (P = 0.80)

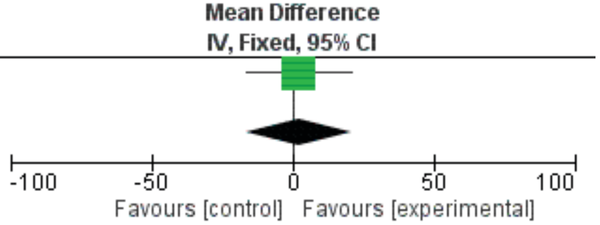

Forest plot of comparison: Amphetamine After Stroke, outcome: Early Motor Performance Month 1.

Figure 2 (Analysis 1.2)

Study or Subgroup	Std. Mean Difference	SE	Weight	Std. Mean Difference IV, Fixed, 95% CI
SONDE 2001	-0.1035	0.3341	52.1%	-0.10 [-0.76, 0.55]
SPRIGG 2007	0.0845	0.3485	47.9%	0.08 [-0.60, 0.77]
**Total (95% CI)**			**100.0%**	**-0.01 [-0.49, 0.46]**

Heterogeneity: Chi² = 0.15, df = 1 (P = 0.70); I² = 0%
Test for overall effect: Z = 0.06 (P = 0.96)

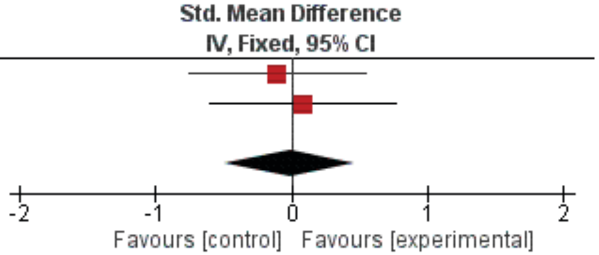

Forest plot of comparison: Amphetamine After Stroke, outcome: Early Motor Performance Month 3.

Figure 3 (Analysis 1.3)

Study or Subgroup	Experimental Mean	Experimental SD	Experimental Total	Control Mean	Control SD	Control Total	Weight	Mean Difference IV, Fixed, 95% CI
SPRIGG 2007	39.1	14.3	17	40.2	12.5	16	100.0%	-1.10 [-10.25, 8.05]
**Total (95% CI)**			**17**			**16**	**100.0%**	**-1.10 [-10.25, 8.05]**

Heterogeneity: Not applicable
Test for overall effect: Z = 0.24 (P = 0.81)

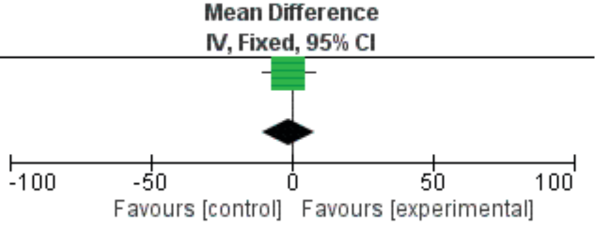

Forest plot of comparison: Amphetamine After Stroke, outcome: Neurological Function Month 1.

Figure 4 (Analysis 1.4)

Study or Subgroup	Experimental Mean	Experimental SD	Experimental Total	Control Mean	Control SD	Control Total	Weight	Mean Difference IV, Fixed, 95% CI
SPRIGG 2007	42.9	11.8	17	41.1	13.8	16	100.0%	1.80 [-6.99, 10.59]
**Total (95% CI)**			**17**			**16**	**100.0%**	**1.80 [-6.99, 10.59]**

Heterogeneity: Not applicable
Test for overall effect: Z = 0.40 (P = 0.69)

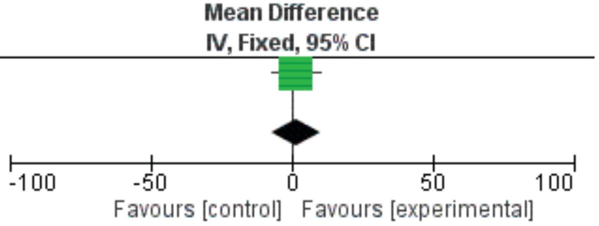

Forest plot of comparison: Amphetamine After Stroke, outcome: Neurological Function Month 3.

Figure 5 (Analysis 1.5)

Study or Subgroup	Experimental Mean	Experimental SD	Experimental Total	Control Mean	Control SD	Control Total	Weight	Mean Difference IV, Fixed, 95% CI
SPRIGG 2007	12.5	5.8	17	11.5	7.3	16	100.0%	1.00 [-3.52, 5.52]
**Total (95% CI)**			**17**			**16**	**100.0%**	**1.00 [-3.52, 5.52]**

Heterogeneity: Not applicable
Test for overall effect: Z = 0.43 (P = 0.66)

Mean Difference IV, Fixed, 95% CI

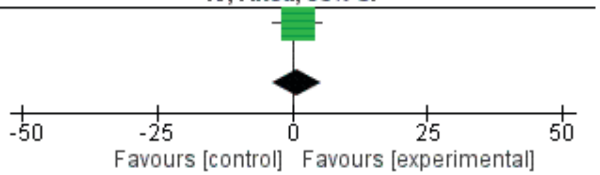

Forest plot of comparison: Amphetamine After Stroke, outcome: Global Functional Outcome Month 1.

Figure 6 (Analysis 1.6)

Study or Subgroup	Experimental Mean	Experimental SD	Experimental Total	Control Mean	Control SD	Control Total	Weight	Mean Difference IV, Fixed, 95% CI
SONDE 2001	69.1	27.9	17	77.1	20.8	19	8.7%	-8.00 [-24.23, 8.23]
SPRIGG 2007	14.3	7.3	17	12.9	7.4	16	91.3%	1.40 [-3.62, 6.42]
**Total (95% CI)**			**34**			**35**	**100.0%**	**0.58 [-4.22, 5.37]**

Heterogeneity: Chi² = 1.18, df = 1 (P = 0.28); I² = 15%
Test for overall effect: Z = 0.24 (P = 0.81)

Mean Difference IV, Fixed, 95% CI

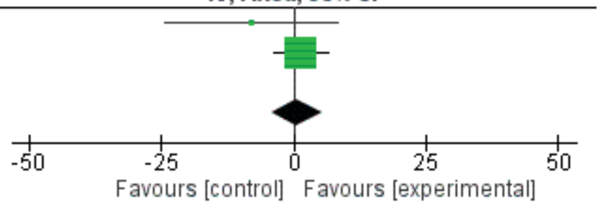

Forest plot of comparison: Amphetamine After Stroke, outcome: Global Functional Outcome Month 3.

Figure 7 (Analysis 1.7)

Study or Subgroup	Experimental Events	Experimental Total	Control Events	Control Total	Weight	Peto Odds Ratio Peto, Fixed, 95% CI
SONDE 2001	0	17	0	19		Not estimable
SPRIGG 2007	3	17	4	16	100.0%	0.65 [0.13, 3.38]
**Total (95% CI)**		**34**		**35**	**100.0%**	**0.65 [0.13, 3.38]**
Total events	3		4			

Heterogeneity: Not applicable
Test for overall effect: Z = 0.51 (P = 0.61)

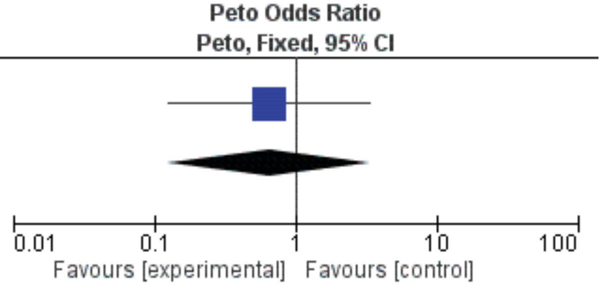

Forest plot of comparison: Amphetamine After Stroke, outcome: Serious Adverse

Events. **Figure 8 (Analysis 2.1)**

Study or Subgroup	Std. Mean Difference	SE	Control Total	Experimental Total	Weight	Std. Mean Difference IV, Fixed, 95% CI
CARS-1 2016	0.5035	0.1535	101	102	100.0%	0.50 [0.20, 0.80]
**Total (95% CI)**			**101**	**102**	**100.0%**	**0.50 [0.20, 0.80]**

Heterogeneity: Not applicable
Test for overall effect: Z = 3.28 (P = 0.001)

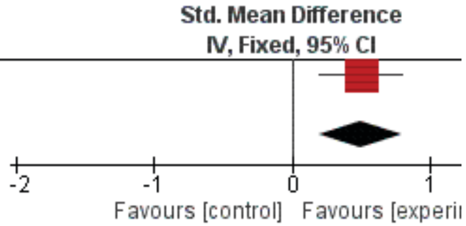

Forest plot of comparison: Cerebrolysin After Stroke, outcome: Early Motor Performance Month 1.

Figure 9 (Analysis 2.2)

Study or Subgroup	Std. Mean Difference	SE	Control Total	Experimental Total	Weight	Std. Mean Difference IV, Random, 95% CI
CARS-1 2016	0.7901	0.173	101	104	48.7%	0.79 [0.45, 1.13]
CARS-2 2017	0.1128	0.1329	120	117	51.3%	0.11 [-0.15, 0.37]
**Total (95% CI)**			**221**	**221**	**100.0%**	**0.44 [-0.22, 1.11]**

Heterogeneity: $Tau^2 = 0.21$; $Chi^2 = 9.64$, df = 1 (P = 0.002); $I^2 = 90\%$
Test for overall effect: Z = 1.31 (P = 0.19)

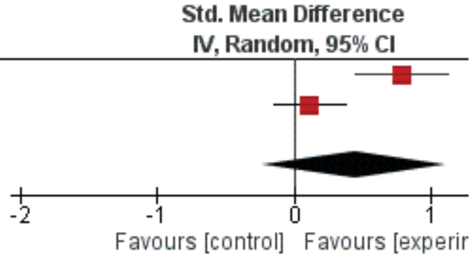

Forest plot of comparison: Cerebrolysin After Stroke, outcome: Early Motor Performance Month 3.

Figure 10 (Analysis 2.3)

Study or Subgroup	Std. Mean Difference	SE	Control Total	Experimental Total	Weight	Std. Mean Difference IV, Random, 95% CI
**2.3.1 Studies with Final Scores**						
AMIRI-NIKPOUR 2014	-0.5236	0.3108	21	22	11.6%	-0.52 [-1.13, 0.09]
**Subtotal (95% CI)**			**21**	**22**	**11.6%**	**-0.52 [-1.13, 0.09]**
Heterogeneity: Not applicable						
Test for overall effect: Z = 1.68 (P = 0.09)						
**2.3.2 Studies with Change Scores**						
CARS-1 2016	-0.3803	0.1466	101	102	36.9%	-0.38 [-0.67, -0.09]
CARS-2 2017	-0.253	0.1326	120	117	41.7%	-0.25 [-0.51, 0.01]
STAN 2017	-0.9588	0.3422	29	30	9.8%	-0.96 [-1.63, -0.29]
**Subtotal (95% CI)**			**250**	**249**	**88.4%**	**-0.40 [-0.68, -0.13]**
Heterogeneity: Tau² = 0.03; Chi² = 3.73, df = 2 (P = 0.15); I² = 46%						
Test for overall effect: Z = 2.84 (P = 0.004)						
**Total (95% CI)**			**271**	**271**	**100.0%**	**-0.40 [-0.62, -0.18]**
Heterogeneity: Tau² = 0.01; Chi² = 3.99, df = 3 (P = 0.26); I² = 25%						
Test for overall effect: Z = 3.55 (P = 0.0004)						
Test for subgroup differences: Chi² = 0.12, df = 1 (P = 0.73), I² = 0%						

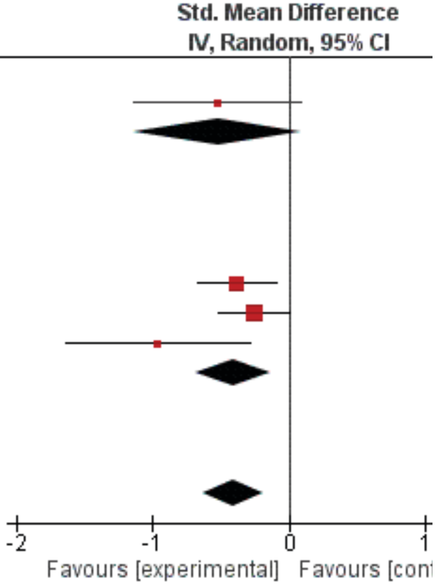

Forest plot of comparison: Cerebrolysin After Stroke, outcome: Neurological Function Month 1.

Figure 11 (Analysis 2.4)

Study or Subgroup	Std. Mean Difference	SE	Control Total	Experimental Total	Weight	Std. Mean Difference IV, Random, 95% CI
**2.4.1 Studies with Final Scores**						
AMIRI-NIKPOUR 2014	-1.0816	0.3287	21	22	28.3%	-1.08 [-1.73, -0.44]
**Subtotal (95% CI)**			**21**	**22**	**28.3%**	**-1.08 [-1.73, -0.44]**
Heterogeneity: Not applicable						
Test for overall effect: Z = 3.29 (P = 0.0010)						
**2.4.2 Studies with Change Scores**						
CARS-1 2016	-0.6433	0.1566	101	104	71.7%	-0.64 [-0.95, -0.34]
**Subtotal (95% CI)**			**101**	**104**	**71.7%**	**-0.64 [-0.95, -0.34]**
Heterogeneity: Not applicable						
Test for overall effect: Z = 4.11 (P < 0.0001)						
**Total (95% CI)**			**122**	**126**	**100.0%**	**-0.77 [-1.15, -0.38]**
Heterogeneity: Tau² = 0.03; Chi² = 1.45, df = 1 (P = 0.23); I² = 31%						
Test for overall effect: Z = 3.89 (P = 0.0001)						
Test for subgroup differences: Chi² = 1.45, df = 1 (P = 0.23), I² = 31.0%						

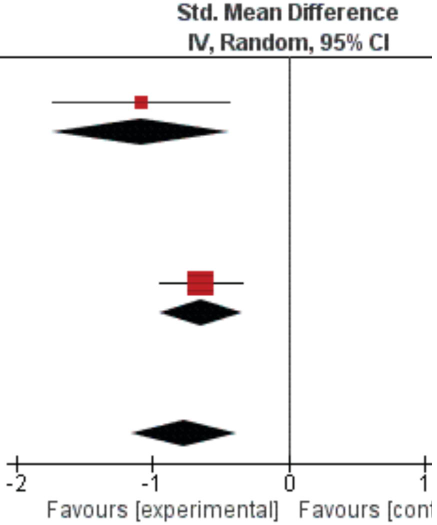

Forest plot of comparison: Cerebrolysin After Stroke, outcome: Neurological Function Month 3.

Figure 12 (Analysis 2.5)

Study or Subgroup	Std. Mean Difference	SE	Control Total	Experimental Total	Weight	Std. Mean Difference IV, Fixed, 95% CI
STAN 2017	-0.8834	0.2944	29	30	100.0%	-0.88 [-1.46, -0.31]
**Total (95% CI)**			**29**	**30**	**100.0%**	**-0.88 [-1.46, -0.31]**
Heterogeneity: Not applicable						
Test for overall effect: Z = 3.00 (P = 0.003)						

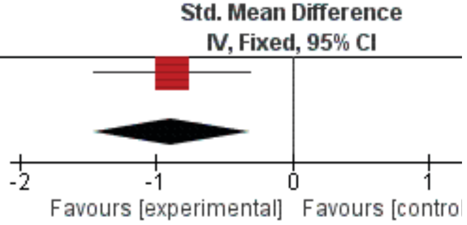

Forest plot of comparison: Cerebrolysin After Stroke, outcome: Global Functional Outcome Month 1.

Figure 13 (Analysis 2.6)

Study or Subgroup	Std. Mean Difference	SE	Control Total	Experimental Total	Weight	Std. Mean Difference IV, Fixed, 95% CI
CARS-1 2016	-0.8834	0.1611	101	104	100.0%	-0.88 [-1.20, -0.57]
**Total (95% CI)**			**101**	**104**	**100.0%**	**-0.88 [-1.20, -0.57]**

Heterogeneity: Not applicable
Test for overall effect: Z = 5.48 (P < 0.00001)

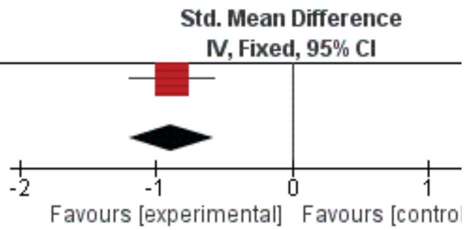

Forest plot of comparison: Cerebrolysin After Stroke, outcome: Global Functional Outcome Month 3.

Figure 14 (Analysis 2.7)

Study or Subgroup	Experimental Events	Experimental Total	Control Events	Control Total	Weight	Peto Odds Ratio Peto, Fixed, 95% CI
CARS-1 2016	3	104	7	104	39.9%	0.43 [0.12, 1.54]
CARS-2 2017	8	120	4	120	47.7%	2.01 [0.63, 6.41]
CHAN 2016	1	35	1	35	8.2%	1.00 [0.06, 16.32]
STAN 2017	0	30	1	30	4.2%	0.14 [0.00, 6.82]
**Total (95% CI)**		**289**		**289**	**100.0%**	**0.92 [0.41, 2.05]**
Total events	12		13			

Heterogeneity: Chi² = 4.03, df = 3 (P = 0.26); I² = 26%
Test for overall effect: Z = 0.20 (P = 0.84)

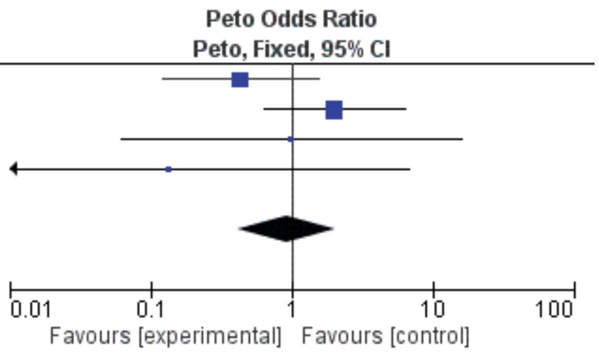

Forest plot of comparison: Cerebrolysin After Stroke, outcome: Serious Adverse Events.

Figure 15 (Analysis 3.1)

Study or Subgroup	Experimental Mean	Experimental SD	Experimental Total	Control Mean	Control SD	Control Total	Weight	Mean Difference IV, Fixed, 95% CI
ACLER 2009	71	10	10	67	10	10	100.0%	4.00 [-4.77, 12.77]
**Total (95% CI)**			**10**			**10**	**100.0%**	**4.00 [-4.77, 12.77]**

Heterogeneity: Not applicable
Test for overall effect: Z = 0.89 (P = 0.37)

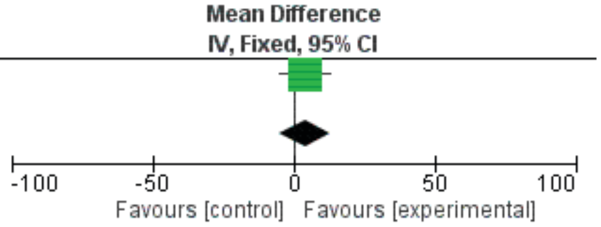

Forest plot of comparison: Citalopram After Stroke (10 mg), outcome: Early Motor Performance Month 1.

Figure 16 (Analysis 3.2)

Study or Subgroup	Experimental Mean	Experimental SD	Experimental Total	Control Mean	Control SD	Control Total	Weight	Mean Difference IV, Fixed, 95% CI
ACLER 2009	2.3	2	10	3.5	1.3	10	100.0%	-1.20 [-2.68, 0.28]
**Total (95% CI)**			**10**			**10**	**100.0%**	**-1.20 [-2.68, 0.28]**

Heterogeneity: Not applicable
Test for overall effect: Z = 1.59 (P = 0.11)

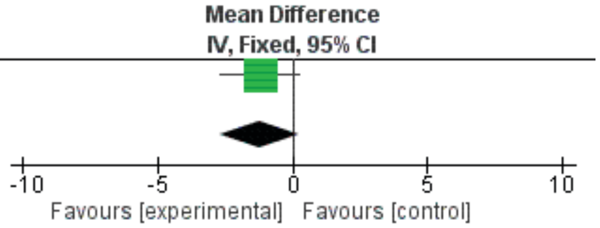

Forest plot of comparison: Citalopram After Stroke (10 mg), outcome: Neurological Function Month 1.

Figure 17 (Analysis 3.3)

	Experimental			Control				Mean Difference
**Study or Subgroup**	**Mean**	**SD**	**Total**	**Mean**	**SD**	**Total**	**Weight**	**IV, Fixed, 95% CI**
ACLER 2009	82	28	10	75	25	10	100.0%	7.00 [-16.27, 30.27]
**Total (95% CI)**			**10**			**10**	**100.0%**	**7.00 [-16.27, 30.27]**

Heterogeneity: Not applicable
Test for overall effect: Z = 0.59 (P = 0.56)

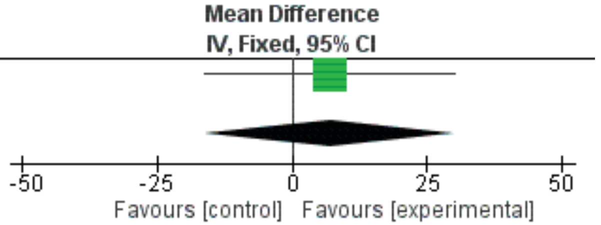

Forest plot of comparison: Citalopram After Stroke (10 mg), outcome: Global Functional Outcome Month 3.

Figure 18 (Analysis 4.1)

	Experimental			Control				Mean Difference
**Study or Subgroup**	**Mean**	**SD**	**Total**	**Mean**	**SD**	**Total**	**Weight**	**IV, Fixed, 95% CI**
OSKOUIE 2017	0.74	0.9	62	1.11	1.01	61	100.0%	-0.37 [-0.71, -0.03]
**Total (95% CI)**			**62**			**61**	**100.0%**	**-0.37 [-0.71, -0.03]**

Heterogeneity: Not applicable
Test for overall effect: Z = 2.14 (P = 0.03)

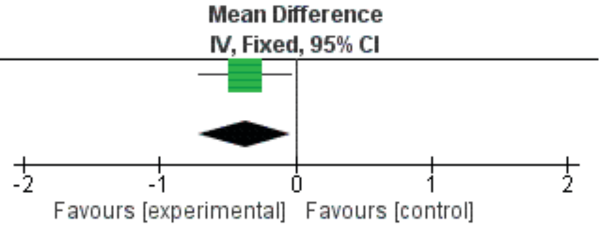

Forest plot of comparison: Citalopram After Stroke (20 mg), outcome: Early Motor Performance Month 1.

Figure 19 (Analysis 4.2)

	Experimental			Control				Mean Difference
Study or Subgroup	Mean	SD	Total	Mean	SD	Total	Weight	IV, Fixed, 95% CI
OSKOUIE 2017	0.25	0.63	62	0.82	0.97	61	100.0%	-0.57 [-0.86, -0.28]
**Total (95% CI)**			**62**			**61**	**100.0%**	**-0.57 [-0.86, -0.28]**

Heterogeneity: Not applicable
Test for overall effect: Z = 3.86 (P = 0.0001)

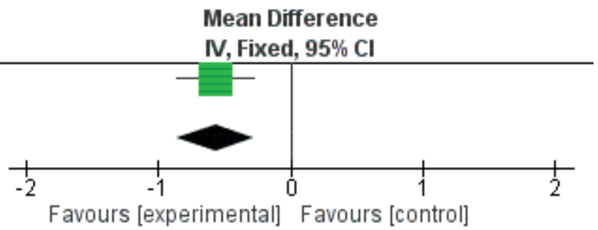

Forest plot of comparison: Citalopram After Stroke (20 mg), outcome: Early Motor Performance Month 3.

Figure 20 (Analysis 4.3)

	Experimental		Control			Risk Ratio
Study or Subgroup	Events	Total	Events	Total	Weight	M-H, Fixed, 95% CI
OSKOUIE 2017	57	72	39	72	100.0%	1.46 [1.15, 1.86]
**Total (95% CI)**		**72**		**72**	**100.0%**	**1.46 [1.15, 1.86]**
Total events	57		39			

Heterogeneity: Not applicable
Test for overall effect: Z = 3.06 (P = 0.002)

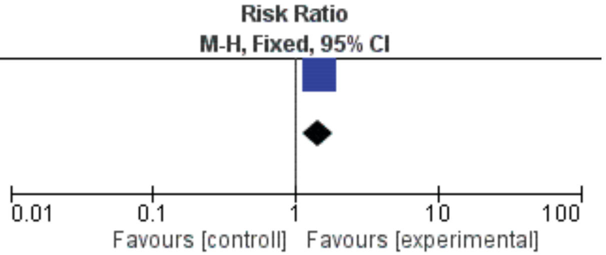

Forest plot of comparison: Citalopram After Stroke (20 mg), outcome: Neurological Function Month 3.

Figure 21 (Analysis 4.4)

Study or Subgroup	Experimental Events	Experimental Total	Control Events	Control Total	Weight	Risk Ratio M-H, Fixed, 95% CI
OSKOUIE 2017	34	59	17	53	100.0%	1.80 [1.15, 2.81]
**Total (95% CI)**		**59**		**53**	**100.0%**	**1.80 [1.15, 2.81]**
Total events	34		17			

Heterogeneity: Not applicable
Test for overall effect: Z = 2.56 (P = 0.01)

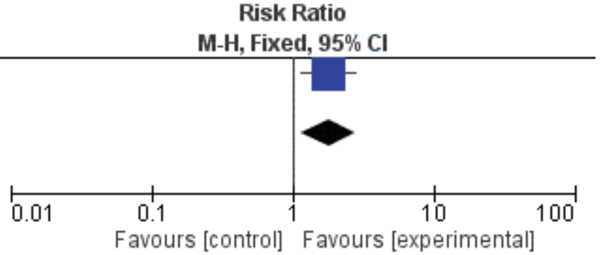

Forest plot of comparison: Citalopram After Stroke (20 mg), outcome: Global Functional Outcome Month 1.

Figure 22 (Analysis 4.5)

Study or Subgroup	Experimental Events	Experimental Total	Control Events	Control Total	Weight	Risk Ratio M-H, Fixed, 95% CI
OSKOUIE 2017	55	58	22	51	100.0%	2.20 [1.60, 3.03]
**Total (95% CI)**		**58**		**51**	**100.0%**	**2.20 [1.60, 3.03]**
Total events	55		22			

Heterogeneity: Not applicable
Test for overall effect: Z = 4.81 (P < 0.00001)

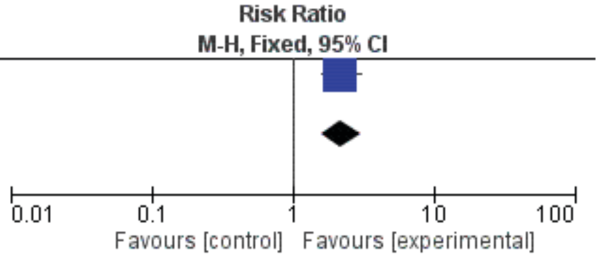

Forest plot of comparison: Citalopram After Stroke (20 mg), outcome: Global Functional Outcome Month 3.

Figure 23 (Analysis 5.1)

	Experimental			Control				Mean Difference
Study or Subgroup	Mean	SD	Total	Mean	SD	Total	Weight	IV, Fixed, 95% CI
GLADSTONE 2005	25.4	15.1	31	24.4	15.9	36	100.0%	1.00 [-6.43, 8.43]
**Total (95% CI)**			**31**			**36**	**100.0%**	**1.00 [-6.43, 8.43]**
Heterogeneity: Not applicable								
Test for overall effect: Z = 0.26 (P = 0.79)								

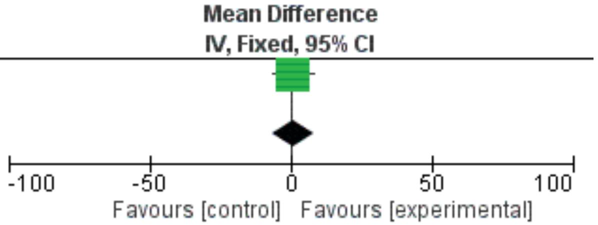

Forest plot of comparison: Dextroamphetamine After Stroke, outcome: Early Motor Performance Month 1.

Figure 24 (Analysis 5.2)

	Experimental			Control				Mean Difference
Study or Subgroup	Mean	SD	Total	Mean	SD	Total	Weight	IV, Fixed, 95% CI
GLADSTONE 2005	29.3	15.7	31	29.7	17.5	36	100.0%	-0.40 [-8.35, 7.55]
**Total (95% CI)**			**31**			**36**	**100.0%**	**-0.40 [-8.35, 7.55]**
Heterogeneity: Not applicable								
Test for overall effect: Z = 0.10 (P = 0.92)								

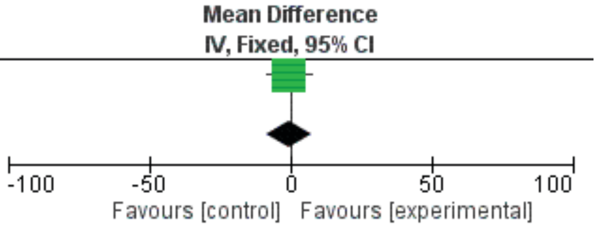

Forest plot of comparison: Dextroamphetamine After Stroke, outcome: Early Motor Performance Month 3.

Figure 25 (Analysis 5.3)

	Experimental			Control				Mean Difference
Study or Subgroup	Mean	SD	Total	Mean	SD	Total	Weight	IV, Fixed, 95% CI
GLADSTONE 2005	29.2	24.5	31	35.4	28.3	36	100.0%	-6.20 [-18.84, 6.44]
**Total (95% CI)**			**31**			**36**	**100.0%**	**-6.20 [-18.84, 6.44]**

Heterogeneity: Not applicable
Test for overall effect: Z = 0.96 (P = 0.34)

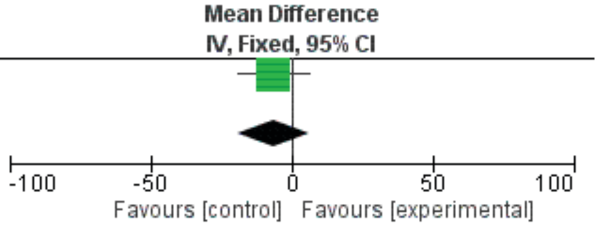

Forest plot of comparison: Dextroamphetamine After Stroke, outcome: Neurological Function Month 1.

Figure 26 (Analysis 5.4)

	Experimental			Control				Mean Difference
Study or Subgroup	Mean	SD	Total	Mean	SD	Total	Weight	IV, Fixed, 95% CI
GLADSTONE 2005	9.8	2.7	31	9.6	2.5	36	100.0%	0.20 [-1.05, 1.45]
**Total (95% CI)**			**31**			**36**	**100.0%**	**0.20 [-1.05, 1.45]**

Heterogeneity: Not applicable
Test for overall effect: Z = 0.31 (P = 0.75)

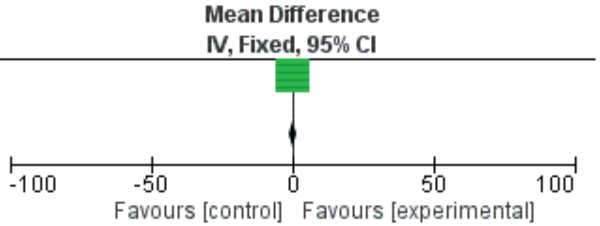

Forest plot of comparison: Dextroamphetamine After Stroke, outcome: Neurological Function Month 3.

Figure 27 (Analysis 5.5)

	Experimental			Control				Mean Difference
**Study or Subgroup**	**Mean**	**SD**	**Total**	**Mean**	**SD**	**Total**	**Weight**	**IV, Fixed, 95% CI**
GLADSTONE 2005	26.1	18.1	31	29.8	15.2	36	100.0%	-3.70 [-11.78, 4.38]
**Total (95% CI)**			**31**			**36**	**100.0%**	**-3.70 [-11.78, 4.38]**
Heterogeneity: Not applicable								
Test for overall effect: Z = 0.90 (P = 0.37)								

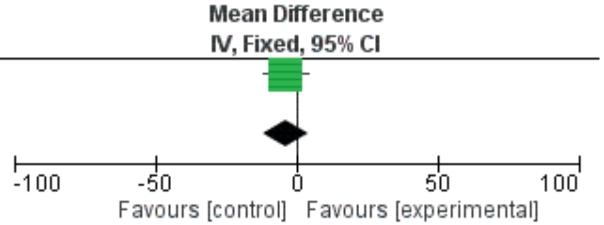

Forest plot of comparison: Dextroamphetamine After Stroke, outcome: Global Functional Outcome Month 1.

Figure 28 (Analysis 5.6)

	Experimental			Control				Mean Difference
**Study or Subgroup**	**Mean**	**SD**	**Total**	**Mean**	**SD**	**Total**	**Weight**	**IV, Fixed, 95% CI**
GLADSTONE 2005	36.9	14.5	31	38.2	17.1	36	100.0%	-1.30 [-8.87, 6.27]
**Total (95% CI)**			**31**			**36**	**100.0%**	**-1.30 [-8.87, 6.27]**
Heterogeneity: Not applicable								
Test for overall effect: Z = 0.34 (P = 0.74)								

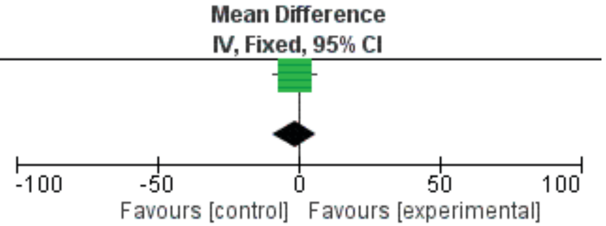

Forest plot of comparison: Dextroamphetamine After Stroke, outcome: Global Functional Outcome Month 3.

Figure 29 (Analysis 6.1)

	Experimental			Control				Mean Difference
**Study or Subgroup**	**Mean**	**SD**	**Total**	**Mean**	**SD**	**Total**	**Weight**	**IV, Fixed, 95% CI**
YU 2015	56.4	12.3155	45	57.6	12.5152	42	100.0%	-1.20 [-6.42, 4.02]
**Total (95% CI)**			**45**			**42**	**100.0%**	**-1.20 [-6.42, 4.02]**

Heterogeneity: Not applicable
Test for overall effect: Z = 0.45 (P = 0.65)

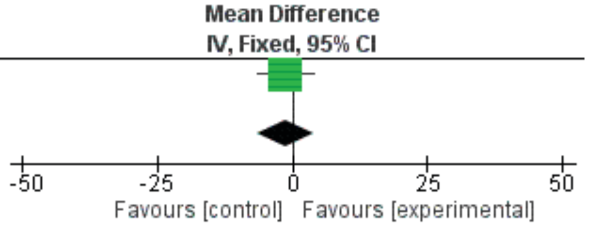

Forest plot of comparison: DHYZ After Stroke, outcome: Early Motor Performance Month 1.

Figure 30 (Analysis 6.2)

	Experimental			Control				Mean Difference
**Study or Subgroup**	**Mean**	**SD**	**Total**	**Mean**	**SD**	**Total**	**Weight**	**IV, Fixed, 95% CI**
YU 2015	71.8	13.9798	45	65.3	13.4779	42	100.0%	6.50 [0.73, 12.27]
**Total (95% CI)**			**45**			**42**	**100.0%**	**6.50 [0.73, 12.27]**

Heterogeneity: Not applicable
Test for overall effect: Z = 2.21 (P = 0.03)

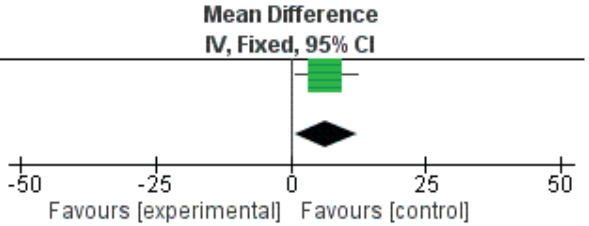

Forest plot of comparison: DHYZ After Stroke, outcome: Early Motor Performance Month 3.

Figure 31 (Analysis 6.3)

	Experimental			Control				Mean Difference
Study or Subgroup	Mean	SD	Total	Mean	SD	Total	Weight	IV, Fixed, 95% CI
YU 2015	52	11.9827	45	55.7	10.2689	42	100.0%	-3.70 [-8.38, 0.98]
**Total (95% CI)**			**45**			**42**	**100.0%**	**-3.70 [-8.38, 0.98]**

Heterogeneity: Not applicable
Test for overall effect: Z = 1.55 (P = 0.12)

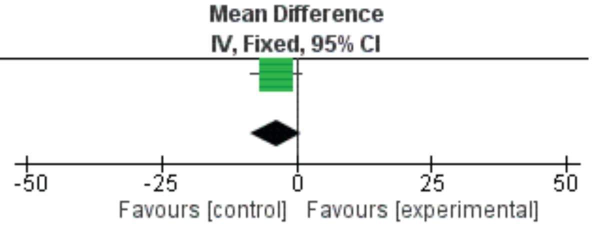

Forest plot of comparison: DHYZ After Stroke, outcome: Global Functional Outcome Month 1.

Figure 32 (Analysis 6.4)

	Experimental			Control				Mean Difference
Study or Subgroup	Mean	SD	Total	Mean	SD	Total	Weight	IV, Fixed, 95% CI
YU 2015	74.6	9.9856	45	70.1	10.2689	42	100.0%	4.50 [0.24, 8.76]
**Total (95% CI)**			**45**			**42**	**100.0%**	**4.50 [0.24, 8.76]**

Heterogeneity: Not applicable
Test for overall effect: Z = 2.07 (P = 0.04)

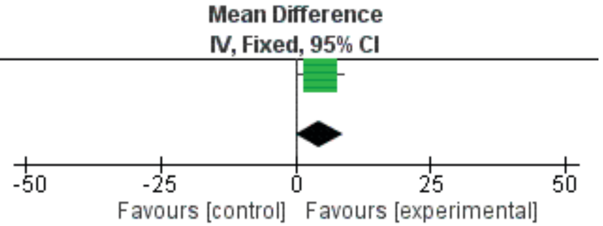

Forest plot of comparison: DHYZ After Stroke, outcome: Global Functional Outcome Month 3.

Figure 33 (Analysis 6.5)

Study or Subgroup	Experimental Events	Experimental Total	Control Events	Control Total	Weight	Peto Odds Ratio Peto, Fixed, 95% CI
YU 2015	0	50	0	50		Not estimable
**Total (95% CI)**		**50**		**50**		**Not estimable**
Total events	0		0			
Heterogeneity: Not applicable						
Test for overall effect: Not applicable						

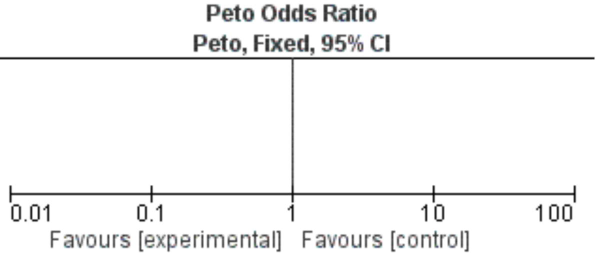

Forest plot of comparison: DHYZ After Stroke, outcome: Serious Adverse Events.

Figure 34 (Analysis 7.1)

Study or Subgroup	Experimental Mean	Experimental SD	Experimental Total	Control Mean	Control SD	Control Total	Weight	Mean Difference IV, Fixed, 95% CI
FLAME 2011	34	16.2059	57	24.3	16.4301	56	100.0%	9.70 [3.68, 15.72]
**Total (95% CI)**			**57**			**56**	**100.0%**	**9.70 [3.68, 15.72]**
Heterogeneity: Not applicable								
Test for overall effect: Z = 3.16 (P = 0.002)								

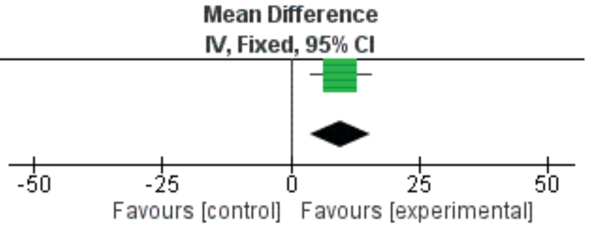

Forest plot of comparison: Fluoxetine After Stroke, outcome: Early Motor Performance Month 3.

Figure 35 (Analysis 7.2)

	Experimental			Control				Mean Difference
**Study or Subgroup**	**Mean**	**SD**	**Total**	**Mean**	**SD**	**Total**	**Weight**	**IV, Fixed, 95% CI**
FLAME 2011	5.8	3.7	57	6.9	4.4	55	100.0%	-1.10 [-2.61, 0.41]
**Total (95% CI)**			**57**			**55**	**100.0%**	**-1.10 [-2.61, 0.41]**

Heterogeneity: Not applicable
Test for overall effect: Z = 1.43 (P = 0.15)

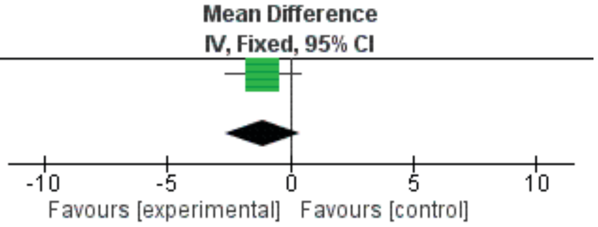

Forest plot of comparison: Fluoxetine After Stroke, outcome: Neurological Function Month 3.

Figure 36 (Analysis 7.3)

**Study or Subgroup**	**Std. Mean Difference**	**SE**	**Weight**	**Std. Mean Difference IV, Fixed, 95% CI**
FLAME 2011	-0.2106	0.1807	100.0%	-0.21 [-0.56, 0.14]
**Total (95% CI)**			**100.0%**	**-0.21 [-0.56, 0.14]**

Heterogeneity: Not applicable
Test for overall effect: Z = 1.17 (P = 0.24)

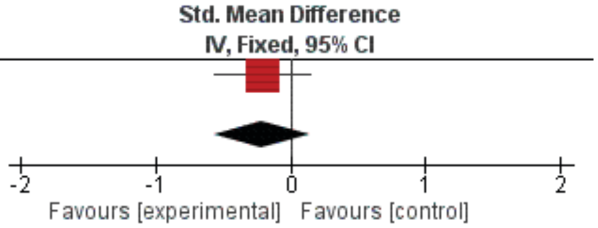

Forest plot of comparison: Fluoxetine After Stroke, outcome: Global Functional Outcome Month 3.

Figure 37 (Analysis 8.1)

	Experimental			Control				Mean Difference
**Study or Subgroup**	**Mean**	**SD**	**Total**	**Mean**	**SD**	**Total**	**Weight**	**IV, Fixed, 95% CI**
MOHAMMADIANINEJAD 2014	2.34	2	32	1.5	2.17	34	100.0%	0.84 [-0.17, 1.85]
**Total (95% CI)**			**32**			**34**	**100.0%**	**0.84 [-0.17, 1.85]**
Heterogeneity: Not applicable								
Test for overall effect: Z = 1.64 (P = 0.10)								

Mean Difference
IV, Fixed, 95% CI

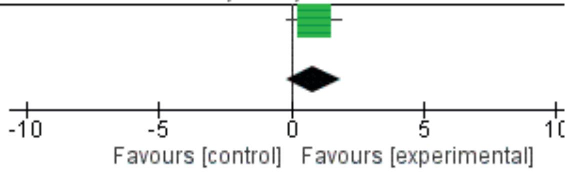

Forest plot of comparison: Lithium After Stroke, outcome: Early Motor Performance Month 1.

Figure 38 (Analysis 8.2)

	Experimental			Control				Mean Difference
**Study or Subgroup**	**Mean**	**SD**	**Total**	**Mean**	**SD**	**Total**	**Weight**	**IV, Fixed, 95% CI**
MOHAMMADIANINEJAD 2014	-2.34	2.13	32	-1.64	1.25	34	100.0%	-0.70 [-1.55, 0.15]
**Total (95% CI)**			**32**			**34**	**100.0%**	**-0.70 [-1.55, 0.15]**
Heterogeneity: Not applicable								
Test for overall effect: Z = 1.62 (P = 0.11)								

Mean Difference
IV, Fixed, 95% CI

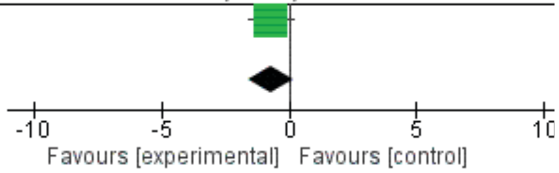

Forest plot of comparison: Lithium After Stroke, outcome: Neurological Function Month 1.

Figure 39 (Analysis 8.3)

Study or Subgroup	Experimental Events	Experimental Total	Control Events	Control Total	Weight	Peto Odds Ratio Peto, Fixed, 95% CI
MOHAMMADIANINEJAD 2014	0	32	0	34		Not estimable
**Total (95% CI)**		**32**		**34**		**Not estimable**
Total events	0		0			
Heterogeneity: Not applicable						
Test for overall effect: Not applicable						

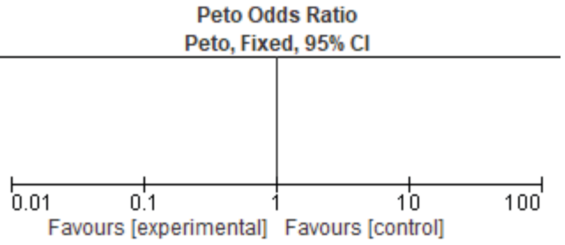

Forest plot of comparison: Lithium After Stroke, outcome: Serious Adverse Events.

Figure 40 (Analysis 9.1)

Study or Subgroup	Mean Difference	SE	Weight	Mean Difference IV, Fixed, 95% CI
CHIMES 2013	-0.17	0.1939	100.0%	-0.17 [-0.55, 0.21]
**Total (95% CI)**			**100.0%**	**-0.17 [-0.55, 0.21]**
Heterogeneity: Not applicable				
Test for overall effect: Z = 0.88 (P = 0.38)				

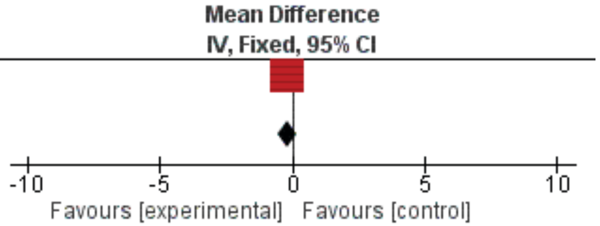

Forest plot of comparison: MLC601 After Stroke, outcome: Early Motor Performance Month 3.

Figure 41 (Analysis 9.2)

Study or Subgroup	Mean Difference	SE	Weight	Mean Difference IV, Fixed, 95% CI
CHIMES 2013	-0.46	0.4082	100.0%	-0.46 [-1.26, 0.34]
**Total (95% CI)**			**100.0%**	**-0.46 [-1.26, 0.34]**

Heterogeneity: Not applicable
Test for overall effect: Z = 1.13 (P = 0.26)

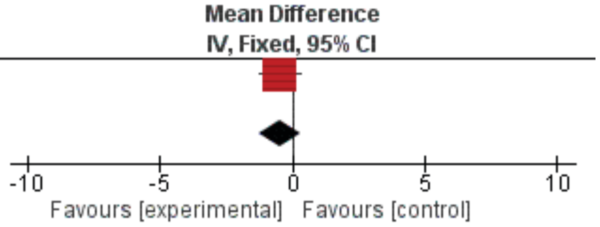

Forest plot of comparison: MLC601 After Stroke, outcome: Neurological Function Month 3.

Figure 42 (Analysis 9.3)

Study or Subgroup	Std. Mean Difference	SE	Weight	Std. Mean Difference IV, Fixed, 95% CI
CHIMES 2013	-0.036162	0.7027092	100.0%	-0.04 [-1.41, 1.34]
**Total (95% CI)**			**100.0%**	**-0.04 [-1.41, 1.34]**

Heterogeneity: Not applicable
Test for overall effect: Z = 0.05 (P = 0.96)

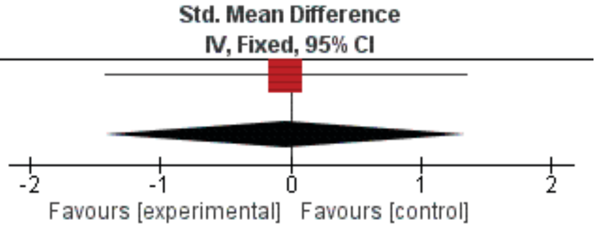

Forest plot of comparison: MLC601 After Stroke, outcome: Global Functional Outcome Month 3.

Figure 43 (Analysis 9.4)

Study or Subgroup	Experimental Events	Experimental Total	Control Events	Control Total	Weight	Peto Odds Ratio Peto, Fixed, 95% CI
CHIMES 2013	60	542	74	545	100.0%	0.79 [0.55, 1.14]
**Total (95% CI)**		**542**		**545**	**100.0%**	**0.79 [0.55, 1.14]**
Total events	60		74			
Heterogeneity: Not applicable						
Test for overall effect: Z = 1.26 (P = 0.21)						

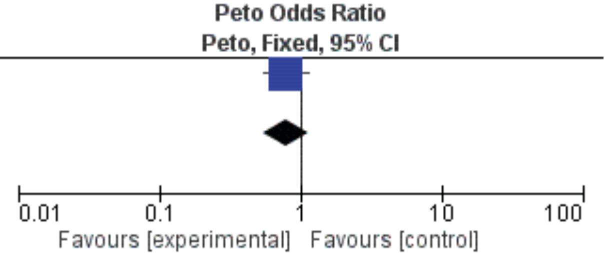

Forest plot of comparison: MLC601 After Stroke, outcome: Serious Adverse Events.

Figure 44 (Analysis 10.1)

Study or Subgroup	Experimental Events	Experimental Total	Control Events	Control Total	Weight	Risk Difference M-H, Fixed, 95% CI
CESARE 2016	17	68	17	65	100.0%	-0.01 [-0.16, 0.14]
**Total (95% CI)**		**68**		**65**	**100.0%**	**-0.01 [-0.16, 0.14]**
Total events	17		17			
Heterogeneity: Not applicable						
Test for overall effect: Z = 0.15 (P = 0.88)						

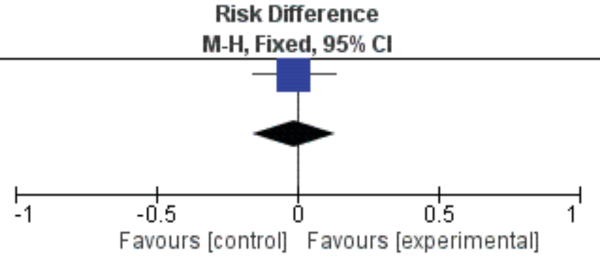

Forest plot of comparison: PF-03049423 After Stroke, outcome: Neurological Function Month 3.

Figure 45 (Analysis 10.2)

Study or Subgroup	Std. Mean Difference	SE	Weight	Std. Mean Difference IV, Fixed, 95% CI
CESARE 2016	-0.1089363	0.17417548	100.0%	-0.11 [-0.45, 0.23]
**Total (95% CI)**			**100.0%**	**-0.11 [-0.45, 0.23]**

Heterogeneity: Not applicable
Test for overall effect: Z = 0.63 (P = 0.53)

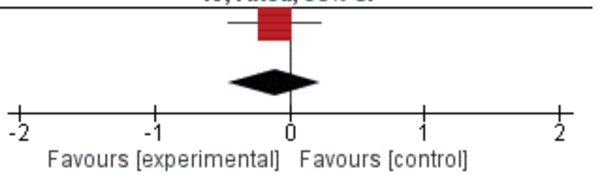

Forest plot of comparison: PF-03049423 After Stroke, outcome: Global Functional Outcome Month 3.

Figure 46 (Analysis 10.3)

Study or Subgroup	Experimental Events	Experimental Total	Control Events	Control Total	Weight	Peto Odds Ratio Peto, Fixed, 95% CI
CESARE 2016	15	70	18	67	100.0%	0.74 [0.34, 1.62]
**Total (95% CI)**		**70**		**67**	**100.0%**	**0.74 [0.34, 1.62]**
Total events	15		18			

Heterogeneity: Not applicable
Test for overall effect: Z = 0.74 (P = 0.46)

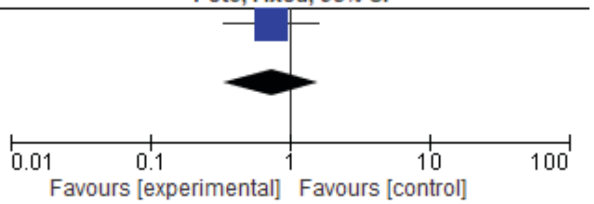

Forest plot of comparison: PF-03049423 After Stroke, outcome: Serious Adverse Events.

Figure 47 (Analysis 11.1)

	Experimental			Control				Mean Difference
Study or Subgroup	Mean	SD	Total	Mean	SD	Total	Weight	IV, Fixed, 95% CI
SIVENIUS 2001	76.1	32.1	9	88.9	20.3	10	100.0%	-12.80 [-37.26, 11.66]
**Total (95% CI)**			**9**			**10**	**100.0%**	**-12.80 [-37.26, 11.66]**

Heterogeneity: Not applicable
Test for overall effect: Z = 1.03 (P = 0.30)

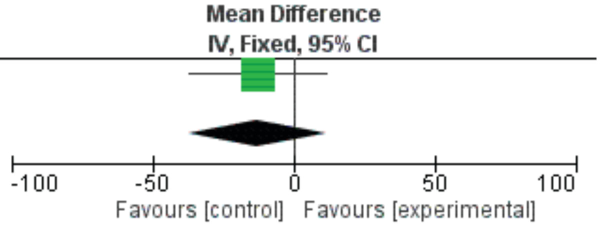

Forest plot of comparison: Selegiline After Stroke, outcome: Early Motor Performance Month 1.

Figure 48 (Analysis 11.2)

	Experimental			Control				Mean Difference
Study or Subgroup	Mean	SD	Total	Mean	SD	Total	Weight	IV, Fixed, 95% CI
SIVENIUS 2001	95.3	16.7	7	93.3	21.2	8	100.0%	2.00 [-17.21, 21.21]
**Total (95% CI)**			**7**			**8**	**100.0%**	**2.00 [-17.21, 21.21]**

Heterogeneity: Not applicable
Test for overall effect: Z = 0.20 (P = 0.84)

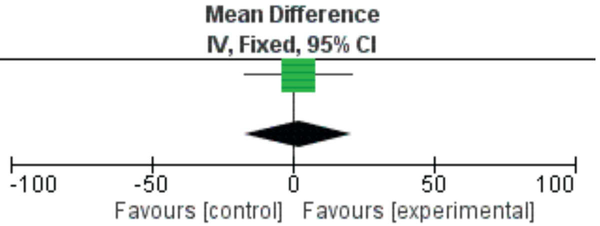

Forest plot of comparison: Selegiline After Stroke, outcome: Early Motor Performance Month 3.

Figure 49 (Analysis 11.3)

Study or Subgroup	Experimental Mean	Experimental SD	Experimental Total	Control Mean	Control SD	Control Total	Weight	Mean Difference IV, Fixed, 95% CI
SIVENIUS 2001	45.6	9.2	9	48.9	7.6	10	100.0%	-3.30 [-10.94, 4.34]
**Total (95% CI)**			**9**			**10**	**100.0%**	**-3.30 [-10.94, 4.34]**

Heterogeneity: Not applicable
Test for overall effect: Z = 0.85 (P = 0.40)

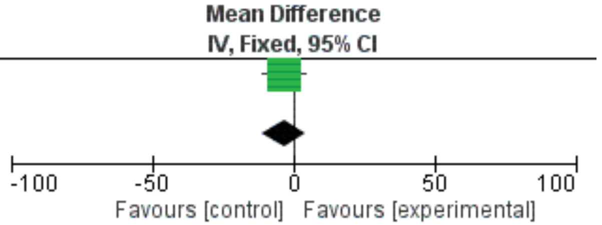

Forest plot of comparison: Selegiline After Stroke, outcome: Neurological Function Month 1.

Figure 50 (Analysis 11.4)

Study or Subgroup	Experimental Mean	Experimental SD	Experimental Total	Control Mean	Control SD	Control Total	Weight	Mean Difference IV, Fixed, 95% CI
SIVENIUS 2001	54.1	3.4	7	51.5	5.2	8	100.0%	2.60 [-1.80, 7.00]
**Total (95% CI)**			**7**			**8**	**100.0%**	**2.60 [-1.80, 7.00]**

Heterogeneity: Not applicable
Test for overall effect: Z = 1.16 (P = 0.25)

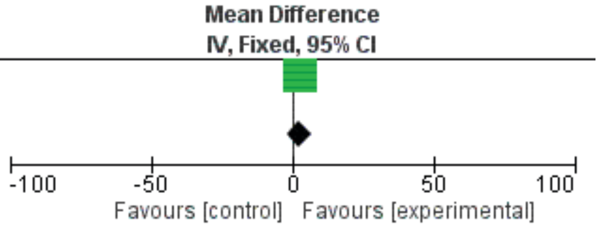

Forest plot of comparison: Selegiline After Stroke, outcome: Neurological Function Month 3.

Figure 51 (Analysis 11.5)

Study or Subgroup	Experimental Mean	Experimental SD	Experimental Total	Control Mean	Control SD	Control Total	Weight	Mean Difference IV, Fixed, 95% CI
SIVENIUS 2001	63.3	33.5	9	66.6	32.4	10	100.0%	-3.30 [-33.00, 26.40]
**Total (95% CI)**			**9**			**10**	**100.0%**	**-3.30 [-33.00, 26.40]**

Heterogeneity: Not applicable
Test for overall effect: Z = 0.22 (P = 0.83)

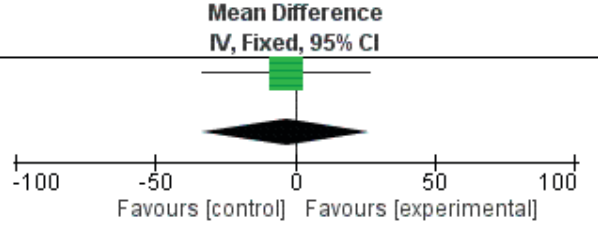

Forest plot of comparison: Selegiline After Stroke, outcome: Global Functional Outcome Month 1.

Figure 52 (Analysis 11.6)

Study or Subgroup	Experimental Mean	Experimental SD	Experimental Total	Control Mean	Control SD	Control Total	Weight	Mean Difference IV, Fixed, 95% CI
SIVENIUS 2001	85.9	20.9	7	80.5	23	8	100.0%	5.40 [-16.82, 27.62]
**Total (95% CI)**			**7**			**8**	**100.0%**	**5.40 [-16.82, 27.62]**

Heterogeneity: Not applicable
Test for overall effect: Z = 0.48 (P = 0.63)

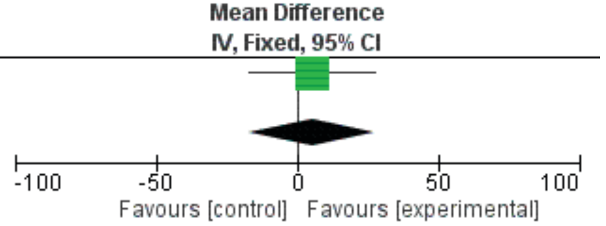

Forest plot of comparison: Selegiline After Stroke, outcome: Global Functional Outcome Month 3.
